# Editorial: Low-Dimension Sensing Nanomaterials

**DOI:** 10.3389/fchem.2021.608327

**Published:** 2021-04-12

**Authors:** Ming-Shui Yao, Wei-Wei Wu, Wen Zeng, Jian-Dong Pang, Jia-Qiang Xu

**Affiliations:** ^1^ Institute for Integrated Cell-Material Sciences, Kyoto University Institute for Advanced Study, Kyoto University, Kyoto, Japan; ^2^ School of Advanced Materials and Nanotechnology, Interdisciplinary Research Center of Smart Sensors, Xidian University, Shaanxi, China; ^3^ College of Materials Science and Engineering, Chongqing University, Chongqing, China; ^4^ Texas A and M University, College Station, TX, United States; ^5^ Department of Chemistry, College of Science, Shanghai University, Shanghai, China

**Keywords:** low-dimension, sensor, nanomaterials, data processing, theoretical calculations

**Editorial on the Research Topic**

**Low-Dimension Sensing Nanomaterials**

Sensing is the art of seeing things invisible (Fourkas, 2011; Yao et al., 2021). Specifically, sensors help human beings collect chemical/physical/bio-stimulus or variations in the environment, and transduce them into readable signals that can be further processed and analyzed (Hu W. et al., 2019; Broza et al., 2019; Xie et al.). In the last 2 decades, the intensive studies on low dimension nanomaterials have indicated the advantages of such materials on developing new sensors with ultrahigh sensitivity, specificity, low-power consumption, multi-functionality, and miniaturized size (Choi and Kim, 2018; Ge et al.; Meng et al., 2019; Qiu and Tang, 2020; Yang et al., 2020).

The Frontiers research topic “Low-Dimension Sensing Nanomaterials” comprises a collection of original research and review articles dealing with the synthesis, chemistry, and applications of low-dimension sensing nanomaterials. This special issue consists of 15 research articles and five mini-reviews, reflecting recent developments on sensing materials, data processing, and theoretical calculations. The collected papers cover the broad applications of sensors in fields of environmental monitoring/cleaning (Li B. et al.; Dong et al.; Luo et al.; Tang et al.), disease diagnosis (Cai et al.; Wang H. et al.), biosensing (Song et al.; Wang Z. et al.), public security affairs (Li Y. et al.), *etc*.

The rich history of sensing materials has made it possible to predict with a relatively high degree of confidence what sensing performances might result if certain well-studied materials are used individually or jointly. However, there are still many worthwhile directions to be pursued to overcome the difficulty to fulfill the “4S” requirements (i.e., sensitivity (response), selectivity, speed (response/recovery time) and stability) at the same time. Firstly, the newly emerging sensing materials (e.g., metal organic framework/porous coordination polymers (MOF/PCPs), black phosphorus, covalent-organic frameworks (COFs), conducting polymers, Mxenes) or hybrid materials always bring new understanding of what the sensor can or cannot do (Hu Y. et al.; Cai et al.; Zhou et al.; Zhuo et al.), which can be further applied to guide the development of existed sensing materials. Secondly, the boosting developments of characterization techniques triggered various in-depth and fundamental studies on both atomic/nanoscale level structure-properties relationships and in-/*ex-situ* sensing mechanisms characterization (Hu Y. et al.; Shao et al.; Song et al.; Wang H. et al.; Jia et al.; Wu et al.). Its great challenge is how to move forward the qualitative measurements to quantitative measurements, summarize the individual cases into general rules via data processing, and thus guide the design of high performance sensors. Last but not the least, the theoretical calculations have contributed greatly to the understanding of sensing mechanisms in the past few decades (Ji et al., 2019; Zhu et al.). With the fast development of simulation theories and computing power, greater contributions on both confirmation and prediction can be expected.

We hope the contributions here can give the inquisitive reader a primer and a springboard that inspires further new ideas in this exciting and challenging research area, in which many more discoveries will be made.
